# Spatiotemporal Suicide Risk in Germany: A Longitudinal Study 2007–11

**DOI:** 10.1038/s41598-017-08117-4

**Published:** 2017-08-09

**Authors:** Marco Helbich, Paul L. Plener, Sebastian Hartung, Victor Blüml

**Affiliations:** 1 Department of Human Geography and Spatial Planning, Utrecht University, 3584 CS Utrecht, Netherlands; 2 Department of Child and Adolescent Psychiatry and Psychotherapy, University of Ulm, 89075 Ulm, Germany; 3 Statistical State Office of the Free State of Saxony, 01917 Kamenz, Germany; 4 Department of Psychoanalysis and Psychotherapy, Medical University of Vienna, 1090 Vienna, Austria

## Abstract

Despite comprehensive prevention programs in Germany, suicide has been on the rise again since 2007. The underlying reasons and spatiotemporal risk patterns are poorly understood. We assessed the spatiotemporal risk of suicide per district attributable to multiple risk and protective factors longitudinally for the period 2007–11. Bayesian space–time regression models were fitted. The nationwide temporal trend showed an increase in relative risk (RR) of dying from suicide (RR 1.008, 95% credibility intervals (CI) 1.001–1.016), whereas district-specific deviations from the grand trend occurred. Striking patterns of amplified risk emerged in southern Germany. While the number of general practitioners was positively related (RR 1.003, 95% CI 1.000–1.006), income was negatively and non-linearly related with suicide risk, as was population density. Unemployment was associated and showed a marked nonlinearity. Neither depression prevalence nor mental health service supply were related. The findings are vital for the implementation of future suicide prevention programs. Concentrating preventive efforts on vulnerable areas of excess risk is recommended.

## Introduction

Suicide mortality is a public health concern in most developed countries^1, 2^. Germany saw declining suicide mortality from 1991 to 2006, but in 2007 this downward trend reversed^3^. The reasons for this development are poorly understood.

While a multitude of factors (e.g., psychological, socio-demographic characteristics, supply of mental health services) play a role in explaining suicide mortality^4–6^, the complexity of suicide epidemiology is increased even more by spatial and temporal variation in risk, not only among countries^1, 7^ but also within them including urban-rural or even district-specific differences^8–13^. Previous ecological studies focused on place-based disparities for a single point in time by pooling suicide data over several years assuming invariable risk^14–18^. Besides loss of information, temporal aggregation biases risk estimates and fails to address changes in temporal trends^19, 20^. The latter limitation is overcome through the application of spatial models for several time periods^10, 21^. Still, important temporal dependences remain unexplored. Incorporating spatiotemporal variability is not only crucial for valid statistical inference^19, 22^, but may also explain why suicide is not universally attributable to similar risk factors and why their magnitudes differ. Although not systematically studied in suicide epidemiology, space–time models can remedy these shortcomings and identify areas with atypically high or low suicide risk, temporal trends, or a combination of both^23–25^.

Taken together, no ecological study had dealt with suicide risk in Germany. Given that suicide risk varies across space and over time, there is a strong need to implement models that explicitly take space–time dynamics into account. Failure to do so might result in a poorer understanding of suicide etiologies and less effective suicide prevention strategies that do not target areas that have a high spatiotemporal risk. This study addressed these pressing research gaps and answered the following research questions:

- How does suicide risk develop in Germany in 2007–11 and which areas are under excessive risk?
- What area-level risk and protective factors are associated with suicide risk?

To answer these questions, two space-time regression models were applied. While a nation-wide increasing suicide risk over time was hypothesized, some districts were expected to have a higher risk than the nation-wide trend. Further, it was hypothesized that areas with high unemployment, low income, low population density, and high depression prevalence increase suicide risk while mental health infrastructure supply diminish risk. The research outcomes are of importance for policy makers who wish to complement individual prevention strategies with place-based strategies over time.

## Methods

### Study design and data

The study design is longitudinal and the population comprised all annual suicide cases in Germany in 2007–11. Officially recorded suicides (i.e., X60–X84) were extracted from the mortality database in accordance with the International Statistical Classification of Diseases and Related Health Problems (10^th^ revision) classification. Suicide data were provided by the Statistical State Office of the Free State of Saxony. As suicide is a rare event, annual data aggregated to districts (*N* = 402) was obligatory. Districts are an appropriate analysis level to represent spatial variation in suicide mortality while not causing privacy issues in less populated areas.

Selecting area-level determinants related to suicide mortality was guided by literature reviews^2, 4, 5, 26^. To control for annual economic wealth^15^, data on the average annual income per person (in €1,000) was included as a time-varying variable. We considered annual unemployment rates (in %, 2007–11) as a proxy variable for social deprivation through financial loss^27, 28^. Both variables were acquired from the German Federal Statistical Office. Data on health service supply for 2011 were collected, including the numbers of general practitioners, psychiatrists, and psychotherapists per 100,000 persons^14, 29^. The first two variables were drawn from the German Federal Statistical Office, the third from the Central Research Institute of Ambulatory Health Care. Due to minor fluctuations in healthcare provision during 2007–11, these variables were kept temporally constant. As depression promotes suicidal thoughts^26^, we adjusted for the prevalence of depressive disorders (in %, ICD-10: F32.x, F33.x, F34.1) based on ambulatory care claims data per district for the year 2011. Data were provided by the Central Research Institute of Ambulatory Health Care. Finally, urban–rural differences in suicide risk^30^ were considered as annual population densities (people per km², 2007–11) provided by the German Federal Statistical Office. No ethical approval was required, because the study used aggregated data.

### Statistical methods

Besides descriptive statistics, annual suicides rates per 100,000 persons were determined to investigate differences in temporal trends and to describe annual spatiotemporal variations per district. Univariate Moran’s *I* tests characterized annual suicide patterns spatially^11, 31^. Positive scores indicate that similar suicide rates are spatially close by, whereas negative values point to dissimilar suicide rates are close by. Bivariate Moran’s *I* statistics characterized spatial correlations between suicide rates at times *t* and *t* + 1. Interpretations remain similar to the univariate case. Neighbourhood between districts was defined as (row-standardized) first-order queen contiguity. Significance was tested through 9,999 permutations against the null hypothesis of spatial randomness. Nonparametric Spearman’s correlations were computed to preclude covariate multicollinearity.

To identify contributing risk and protective factors from 2007 to 2011 and to investigate spatiotemporal suicide risk, hierarchical Bayesian Poisson models were set up with suicide counts as response^24^. We applied a parametric time trend model^23, 32^ and a non-parametric dynamic model^25^ which were found to be superior to more complex models^33^.

Model 1a has a parametric linear time trend. Whereas the covariate income, unemployment rate, and population density is time-varying, the remaining covariates are kept temporally constant. In model 1b, significantly associated linear covariates are replaced with second-order random walks to explore non-linear effects^16^. To obviate violation of spatial independence between adjacent districts, it was of paramount importance to model area-specific effects, because spatially adjacent districts have an associated risk. A spatially structured residual effect modelled as intrinsic conditional autoregressive specification and an unstructured residual effect were implemented^34^. Districts are neighbours when a common boundary is shared. Rather than pooling data over time, models 1a and 1b consider temporal dependences through a nationwide temporal trend and a differential component captures district-related temporal deviations from the grand trend. A negative differential component indicates a less pronounced trend than the grand trend, and a positive value refers to a more pronounced trend. Model 2 relaxes the linearity assumption of the temporal effect in models 1a and 1b through a second-order random walk and a temporally unstructured effect^23^. The supplementary information provides details about the models.

Bayesian inference was carried out with the integrated nested Laplace approximation (INLA)^35^. Although the default prior distributions were specified for the model parameters, the hyperparameters for the spatial effects and random walks were scaled to achieve a less ad hoc selection. For all models, relative risk estimates were obtained together with the 95% credibility intervals (CI). If the 95% CI does not include one, strong statistical evidence for an association is given. The deviance information criterion (DIC) assessed the goodness-of-fit. Lower DIC values denote better models^24^.

## Results

### Descriptive statistics

**Figure 1:**
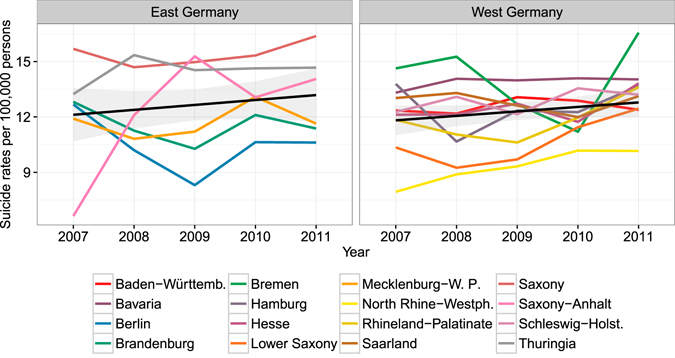
Suicide rates 2007–11 per federal state (the black line shows the temporal trend together with the 95% confidence interval).

A total of 48,570 suicides occurred in 2007–11, with a peak of 10,136 in 2011. The trajectory of the suicide rate shows a constant temporal increase from 11.4 deaths per 100,000 persons in 2007, to 12.6 deaths per 100,000 persons in 2011. This increasing suicide prevalence is less clear when examining the 16 federal states in Fig. 1. Whereas the estimated trend using a generalized additive model still indicates an increase, the individual federal states show more complex patterns. For example, with suicide rates of between 14.7 (2008) and 16.4 (2011) per 100,000 persons, Saxony remained at a high level. In contrast, North Rhine–Westphalia saw an increase from 7.9 (2007) to 10.2 (2011) suicides per 100,000 persons.

Descriptives are provided in Supplementary Table S1. Supplementary Fig. S2 shows the spatial distribution of suicide rates stratified by year. The general trend of increasing suicides on the federal state level translates to the district level. Whereas in 2007 the eastern areas of Saxony, Thuringia, northern Bavaria, and southern Brandenburg show higher rates, this pattern shifted over time. In 2011, a predominance of higher suicide prevalence is apparent in the central and eastern areas (e.g., Saxony, Thuringia) compared to the western parts.

### Exploratory analyses

Multicollinearity among the covariates is not found (see Supplementary Table S3). Univariate Moran’s *I* tests confirm autocorrelated annual suicide rates (see Supplementary Table S4). Whereas the Moran’s *I* values increase from 2007 (*I* = 0.222, *P* = 1e-04) to 2008 (*I* = 0.273, *P* = 1e-04), a decline is apparent until 2011 (*I* = 0.077, *P* = 0.009), signifying a spatially less structured suicide pattern. The bivariate Moran’s *I* statistics between two timestamps confirm that spatial autocorrelation is significantly persistent over time (*P* = 1e-04) (see Supplementary Table S5), namely that similar suicide rates at time *t* are surrounded by similar ones at *t* + 1. These findings are critical for subsequent analyses, as suicide rates are related not only across space but also over time, supporting spatiotemporal regressions.

### Spatiotemporal regressions

Besides the null models (i.e., regressions without covariates), two ecological regressions with increasing complexity were estimated. Lower DIC scores suggest that the ecological regressions are superior to the null models (Table 1). The DIC clearly favours model 1a over model 2 (12,330 vs. 12,352). Model 1a was re-estimated whereby significant linear effects were replaced with non-linear terms (model 1b). The DIC drops to 12,324 referring to improvements in fit. Further discussion deals with model 1b.

**Table 1:** Results of spatiotemporal regressions.

Parametric time trend models							Non-parametric dynamic time trend model		
Model 1a				Model 1b			Model 2		
	RR	2.5% CI	97.5% CI	RR	2.5% CI	97.5% CI	RR	2.5% CI	97.5% CI
Intercept	0.936	0.706	1.242	0.739	0.598	0.914	0.980	0.738	1.302
Year	1.009	1.001	1.017	1.008	1.001	1.016	NLE		
Income (in €1,000)	0.994	0.989	0.999	NLE			0.995	0.990	1.001
Unemployment rate (in %)	1.015	1.007	1.023	NLE			1.016	1.008	1.023
Depression prevalence (in %)	1.010	0.997	1.023	1.009	0.996	1.022	1.008	0.995	1.021
General practitioners (per 100,000)	1.003	1.000	1.006	1.003	1.000	1.006	1.003	1.000	1.006
Psychiatrists (per 100,000)	1.000	0.999	1.002	1.000	0.998	1.002	1.000	0.998	1.002
Psychotherapists (per 100,000)	1.005	0.995	1.015	1.003	0.993	1.013	1.007	0.997	1.016
Population density (logged)	0.959	0.932	0.987	NLE			0.954	0.927	0.982
DIC	12,330			12,324			12,352		
DIC null model	12,334						12,357		

Note: NLE = estimated as non-linear effect, RR = relative risk, CI = credibility intervals.

**Figure 2:**
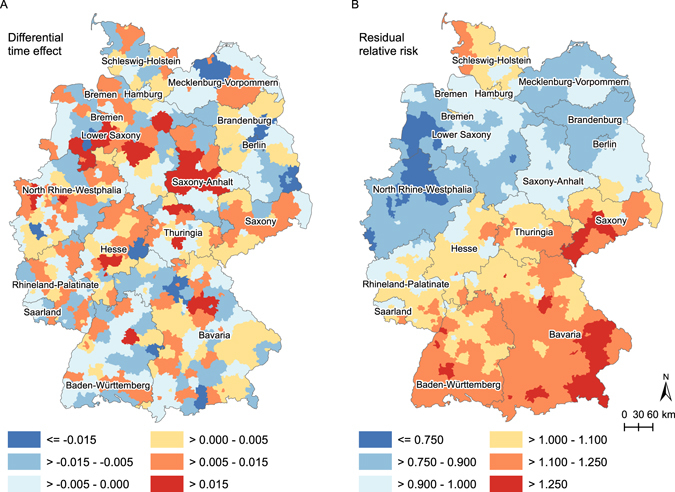
Differential time effect (**A**) and residual relative risk (**B**) per district (model 1b). Maps were created with ArcGIS 10.4.1 (www.esri.com).

Congruent with Fig. 1, the nationwide temporal risk increased over time (RR 1.008, 95% CI 1.001–1.016 (see Supplementary Fig. S6) even after adjusting for the risk and protective factors. District-specific trends deviating from the grand increase are shown in Fig. 2A. Whereas a more distinct upward differential time trend appears in more central areas, areas such as Berlin show a less steep trend. Except for Berlin, the statistical evidence of the differential effects is weak. A distinct spatial pattern in the differential time effect is hardly recognizable, in sharp contrast to the residual relative risk for each district (i.e., exponentiating the sum of the area-specific structured and unstructured effect resulting from model 1b) compared to Germany (Fig. 2B). Districts in the southern half of Germany show a concentration of elevated risk. The more central districts have a lower district-specific residual relative risk. In the northern districts, the risk is elevated again.

**Figure 3:**
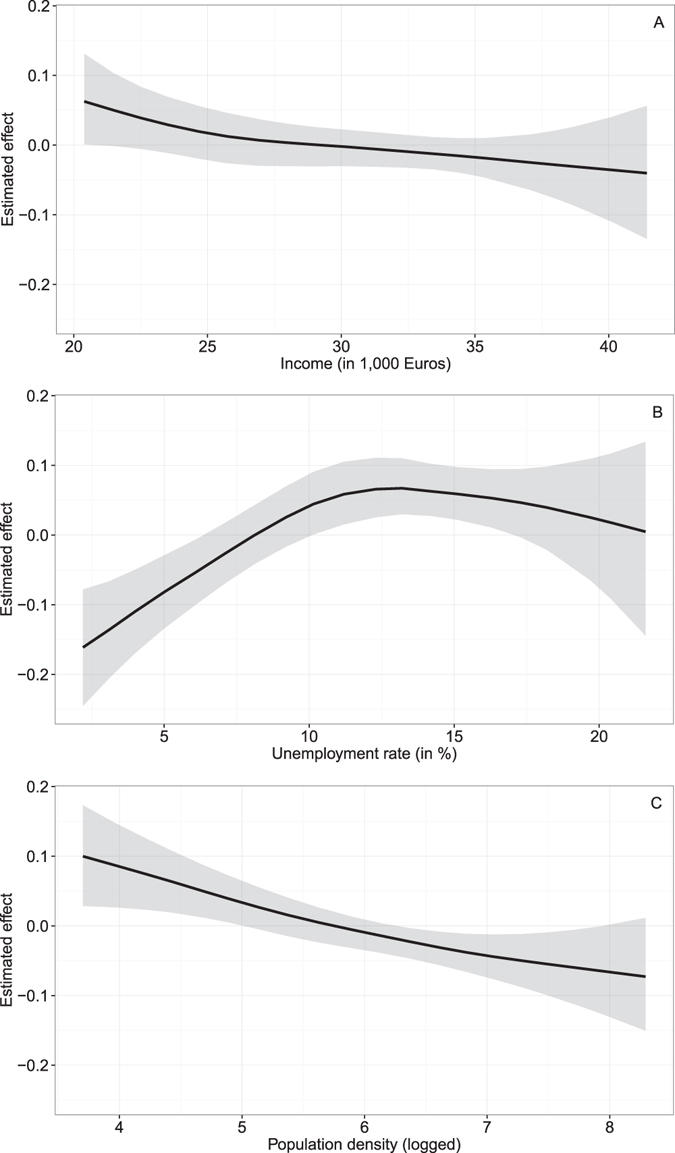
Non-linear risk factors for income (A), unemployment rate (B), and population density (C).

Neither depression prevalence nor the supply of mental health services (i.e., numbers of psychiatrists and psychotherapists per 100,000 people) were associated with suicide risk, as the CIs contain one (Table 1). Supported is a positive correlation of general practitioners per 100,000 persons (RR 1.003, 95% CI 1.000–1.006). Both parametric models provide strong evidence that income (RR 0.994, 95% CI 0.989–0.999) and unemployment rate (RR 1.015, 95% CI 1.007–1.023), both considered as time-varying covariates, are key to explaining risk of suicide. Income has an inverse effect (i.e., the lower income, the higher the relative risk of dying from suicide), whereas unemployment rate has a positive effect (i.e., the higher unemployment rates, the higher the relative risk of suicide). Relaxing the linearity assumption for both covariates (model 1b) resulted in the non-linear relationships displayed in Fig. 3. A slightly more pronounced negative effect is noticeable for lower income areas between €20,000 and €25,000, but in general a linear effect approximates the inverse association well (Fig. 3A). Unemployment rate is, in contrast, non-linearly associated with suicide risk (Fig. 3B). A pronounced positive effect is observable for rates between approximately 1 and 11%. After this peak, the effect flattens out. Logged population density is negatively associated with suicide risk. The association approximates a linear effect (Fig. 3C). Sensitivity tests with changed hyperparameters did not alter the results, confirming the robustness of the results.

## Discussion

As we confirmed for Germany, striking spatial disparities in suicide risk are found^13–16, 36^. The regional variation in suicide risk were similar to those found by an Austrian study^31^. Spatial clustering of suicides might be influenced by amplified cultural acceptance^37^. Similar to a British study^38^ our space–time model revealed changes in suicide risk patterns over time, a phenomenon that has thus far been disregarded^39^. The grand linear upward trend confirmed our hypothesis and is consistent with Hegerl *et al*.^3^. While for Spain a non-linear time effect was reported^22^, we found no support for such a conclusion. M3ore important, some districts show an increase in suicide risk compared to the nationwide trend. To explain the emergence of high risk areas over time, besides sharing similar socio-economic characteristics, several psychological mechanisms are plausible. Among them, contagion suggests that suicide predisposition is sparked by imitation or priming within geographic proximity stimulating suicidal thoughts^5^.

The results concerning risk and protective factors are mixed across studies; possibly due to mediating effects between variables^7^. Although a study using a German population-based sample found null associations^40^, income was negatively associated with suicide mortality. The effect was slightly more pronounced for areas with an average annual income per person of below €25,000. An inverse relationship is congruent with a Danish register-based analysis^41^ and an ecological study for Taiwan^15^. A negative relationship is a result of limited economic opportunities causing less self-confidence while low income areas are economically more deprived, which has detrimental health effects^27^.

In keeping with previous studies^28, 42^, the unemployment rate was positively associated with suicide. Labour market participation constitutes a certain role in society. Material losses and decline in social reputation related to joblessness provoke anxiety and psychological stress, which puts out-of-work people at high risk^37^. Our positive effect confirms an Australian study^42^, but contradicts one for Germany^27^. Circumventing deficits of cross-sectional research designs, a European-wide panel study found that unemployment was a significant attributable risk^28^. In contrast to previous assumptions of a linear association, we showed that the unemployment rate is strongly non-linearly related to suicide risk. The positive relationship peaks at 11%, above which the effect declines. Although speculative, it seems that when a critical mass is reached, people resign themselves to being jobless because others are in a similar situation.

People suffering from depressive disorders have a higher suicide risk^26^. Depression prevalence as a central risk factor is barely considered in area-based studies^21, 31^, but Danish register studies found confirmatory evidence for a positive association^41^. Our results could not replicate these findings and reject our hypothesis. However, our data on depression prevalence reflect diagnosed cases of depression, and do not necessarily capture undiagnosed and therefore untreated cases of depression, which are at even greater risk of suicide. Besides, a meta-analysis for East Asia regions conclude that mental disorders among suicide cases are relatively low^43^.

No evidence was found that the availability of psychiatrists and psychotherapists per 100,000 persons reduces suicide risk due to enhanced diagnoses and treatment opportunities^13, 44^. In Finland and Australia, access to mental health services significantly diminishes risk^14, 29^. The availability of at least one psychiatrist per 100,000 persons has protective effects against death by suicide in Japan^39^. However, in Germany 61% of depressed patients are treated by general practitioners^45^. General practitioners per 100,000 persons showed a weak positive correlation with suicide risk in Austria^44^. Limited training in diagnosing suicidal behaviour and administering effective treatments for at-risk individuals could contribute to this finding^39^. Comparing urban–rural areas showed that general practitioners are more available in rural areas than in cities (mean rural density: 65.3 vs. mean urban density: 64.0); the opposite is true for the mean psychiatrist density: 4.4 vs. 6.2.

We found striking urban-rural differences in that areas with higher population densities have a lower suicide risk. Factors that might contribute to a greater rural suicide risk include limited mental health treatment due to poor access to health infrastructure, reduced interpersonal relationships and thus increased social isolation, and the greater availability of firearms^30^. Significant suicide risk disparities are reported for different degrees of urbanization^13, 21^, although the literature is inconclusive. A pronounced suicide risk in less urbanized areas was found in England and Wales and in Taiwan^10, 15^. For Austria, an inverse association between urbanicity and suicide deaths for men only was reported^13^ a study in Flanders, Belgium, had a similar finding^37^. No significant urban–rural differences in suicide were found for Germany^40^. Caution is advised when cross-comparing results, as the study designs and analyses scales differ, which might lead to conflicting conclusions^6^.

This study was one of the first to explore the space–time patterning of suicide, and is unique in dealing with the German context. Challenging previous studies^10, 21, 36^, a major advantage was gained by using a longitudinal research design. Further, we considered time-varying covariate, non-linearities, and both spatial dependence and temporal trends within a uniform model. This investigation compared different cutting-edge space–time models for suicidology. Another key strength is the rich set of control variables such as proxies for psychiatric treatment and depression prevalence. This minimized residual confounding.

Although we broke new ground, this study had some limitations. Besides the universal shortcoming of ecological research designs, cause–effect relationships cannot be inferred^20, 46^. For this, longitudinal cohort studies at an individual level are recommended. In high-income countries, suicide is more prevalent among elderly males^2^. Due to data privacy, we could not explore fluctuations between gender and age cohorts or differences across suicide methods^47^. Depression prevalence data, limited to one year, comprised all insured patients utilizing psychosocial services covered by public health insurance. Although about 90% of German citizen have public health insurance, confounding due to privately insured patients^48^, unrecognized patients^49^, and limited data validity due to varying depression diagnostic habits cannot be ruled out^50^. Finally, having a short time interval of five years, the full capacity of space-time models might be limited.

In conclusion, this study examined space–time suicide mortality at the district level in Germany in 2007–11. Germany has experienced a substantial upward trend in suicide risk. Some districts deviated from this nationwide trend, facing pronounced risk over time. Striking patterns of elevated risk emerged in southern Germany. Multiple risk and protective factors were identified. Unemployment and a higher density of general practitioners are positive associated with suicidal risk, whereas income and population density are negatively correlated.

The findings have compelling implications for public health policies. While the increase in suicide risk over time calls into question the effectiveness of the country’s suicide prevention programs, efforts to reduce the health burden of suicide (i.e., allocation of financial means, localized health policies) are advised to prioritize vulnerable areas of high spatiotemporal risk. Evidence-based and time–place-specific strategies coupled with well-established treatments of suicidal behaviour at a personal level^51^ seem appropriate to mitigate a downward spiral and prevent excess risk spilling over to adjacent areas.

### Data Availability Statement

The data that support the findings of this study are available from Statistical State Office of the Free State of Saxony but restrictions apply to the availability of these data, which were used under license for the current study, and so are not publicly available. Data are however available from Statistical State Office of the Free State of Saxony upon reasonable request.

## Electronic supplementary material

Supplementary materials
