# Supplementary material for: Spatiotemporal Suicide Risk in Germany: A Longitudinal Study 2007–11
Source: Sci Rep. 2017 Aug 9;7:7673. doi: 10.1038/s41598-017-08117-4 (PMC5550498; doi:10.1038/s41598-017-08117-4)
Supplement: Supplementary file 1 — Supplementary materials [file 41598_2017_8117_MOESM1_ESM.pdf]

Supplementary Materials

Spatiotemporal Suicide Risk in Germany: A Longitudinal Study 2007–11

Marco Helbich, Paul L. Plener, Sebastian Hartung, Victor Blüml

Specification of space–time regressions

To model contributing risk and protective factors from 2007 to 2011 and to investigate spatiotemporal suicide risk, hierarchical Bayesian models were implemented [1,2]. The application was restricted to the parametric time trend model [3] and the non-parametric dynamic counterpart [1,4] which were found to be superior to more complex models [5,6].

As suicide is a count, it is valid to assume that these counts arise from a Poisson distribution. Let y_{it} be the observed suicide cases in area *i* (*i*=1,…,402) at time *t* (*t*=2007,…,2011), ρ_{it} denotes a rate, and E_{it} represent the expected number of cases [1,2]. Then the implemented mode is expressed as (equation 1):

$$y_{it} = \text{Poisson}(\lambda_{it}) \qquad \lambda_{it} = E_{it}\rho_{it} \qquad \log(\rho_{it}) = \eta_{it} \tag{1}$$

Model 1a follows Bernardinelli et al. (1995) who proposed the following spatiotemporal model with a parametric linear time trend (equation 2):

$$\eta_{it} = \alpha + \beta_k x_{kt} + v_i + u_i + (\psi + \delta_i) \times t \tag{2}$$

where the intercept α represents the area-wide relative risk and β_k refers to the regression coefficient of covariate *k*. Whereas the covariate income, unemployment rate, and population density is time-varying, the remaining covariates are kept temporally constant. In model 1b, significantly associated linear covariates are replaced through second-order random walks to model non-linear effects [7,8]. To obviate violation of the model assumption of spatial independence, it was of paramount importance to explicitly model area-specific spatial effects; that is, spatially adjacent districts have an associated risk. Here, following Besag et al. (1991), v_i is a spatially structured residual effect for each district modeled as intrinsic conditional autoregressive specification. An unstructured residual effect u_i

models spatially uncorrelated extra variability provoked by unobserved aspatial variables and is assumed to follow a Gaussian distribution [10]. The smoothed district-specific relative risk compared to entire Germany is $\zeta_i = \exp(v_i + u_i)$. Districts are neighbours when a common boundary is shared. Rather than pooling data over time, models 1a and 1b consider temporal dependencies. ψ represents the grand (i.e., study area-wide) temporal trend, whereas the differential component, δ_i, captures district-related temporal deviations modelled as independent and Gaussian distributed random variable. Interpretatively, a differential component below 0 indicates a less pronounced trend than the grand trend, and a value of above 0 refers to a more pronounced trend [2].

Model 2 relaxes the linearity assumption of the temporal effect in models 1a and 1b by means of a nonparametric dynamic time trend [4] (equation 3):

$$\eta_{it} = \alpha + \beta_k x_{kt} + v_i + u_i + \gamma_t + \phi_t \qquad (3)$$

Here, the same parametrization applies as above except that γ_t now refers to a temporally structured effect modelled as second-order random walk and ϕ_t to a temporally unstructured effect. The temporally structured effect aims to resemble the trend of adjacent districts.

Bayesian inference was carried out with the integrated nested Laplace approximation, which is a highly accurate and computationally fast alternative to Markov chain Monte Carlo methods [11]. Although the default prior distributions were specified for the model parameters, the hyperparameters for the spatial effects and random walks were scaled to achieve a less ad hoc selection [12]. For all models, relative risk estimates were obtained based on exponentiating their posterior means together with the 95% credibility intervals (CI) to obtain parameter uncertainty. In order to assess the quality of the models, three statistics were utilized: a) The deviance information criterion (DIC) to judge the goodness-of-fit and to compare candidate models [13]. Lower DIC values denote a better trade-off between a model’s performance and its complexity. b) The sum of the logged cross-validation predictive ordinate values (CPO) [14]. Larger scores refer to a better model fit. c) The predictive quality, which was tested through histograms of the probability integral transform (PIT). Uniform distribution of the PIT histogram indicates a well-specified model. CPO and PIT results are available upon request.

Supplementary Table S1. Descriptive statistics.

	Min.	1st Qu.	Median	3rd Qu.	Max.
Suicides					
2007	0	12	18	27	433
2008	0	12	19	27	350
2009	0	12	19	28	286
2010	0	13	19	29	368
2011	0	13	19	30	353
Population					
2007	34,719	106,738	150,131	204,522	3,416,255
2008	34,525	105,892	149,442	238,840	3,431,675
2009	34,109	105,597	148,602	238,204	3,442,675
2010	33,944	105,211	148,629	238,891	3,460,725
2011	34,161	104,350	147,000	237,796	3,326,002
Income (in €)					
2007	19,934	23,552	25,966	27,898	38,637
2008	19,892	23,770	26,537	28,520	38,715
2009	19,649	23,996	26,546	28,280	39,389
2010	20,130	24,591	27,235	29,098	39,420
2011	20,573	25,370	28,000	30,080	41,864
Population density (people per km²)					
2007	40	117	204	651	4,100
2008	39	116	203	649	4,140
2009	39	115	201	649	4,150
2010	38	114	200	649	4,230
2011	37	113	196	638	4,260
Unemployment rate (in %)					
2007	2.2	5.3	7.4	11.1	22.0
2008	1.6	4.3	6.4	9.6	19.4
2009	2.2	4.9	6.9	9.9	18.0
2010	1.9	4.7	6.5	9.2	16.6
2011	1.4	4.0	5.9	8.5	16.7
Depression prevalence 2011 (in %)	5.3	8.8	9.9	11.2	18.2
General practitioners 2011 (per 100,000 persons)	46.1	59.3	64.0	68.2	96.6
Psychiatrists 2011 (per 100,000 persons)	0.0	3.4	4.3	6.1	19.3
Psychotherapists 2011 (per 100,000 persons)	1.7	10.9	16.1	24.8	129.8

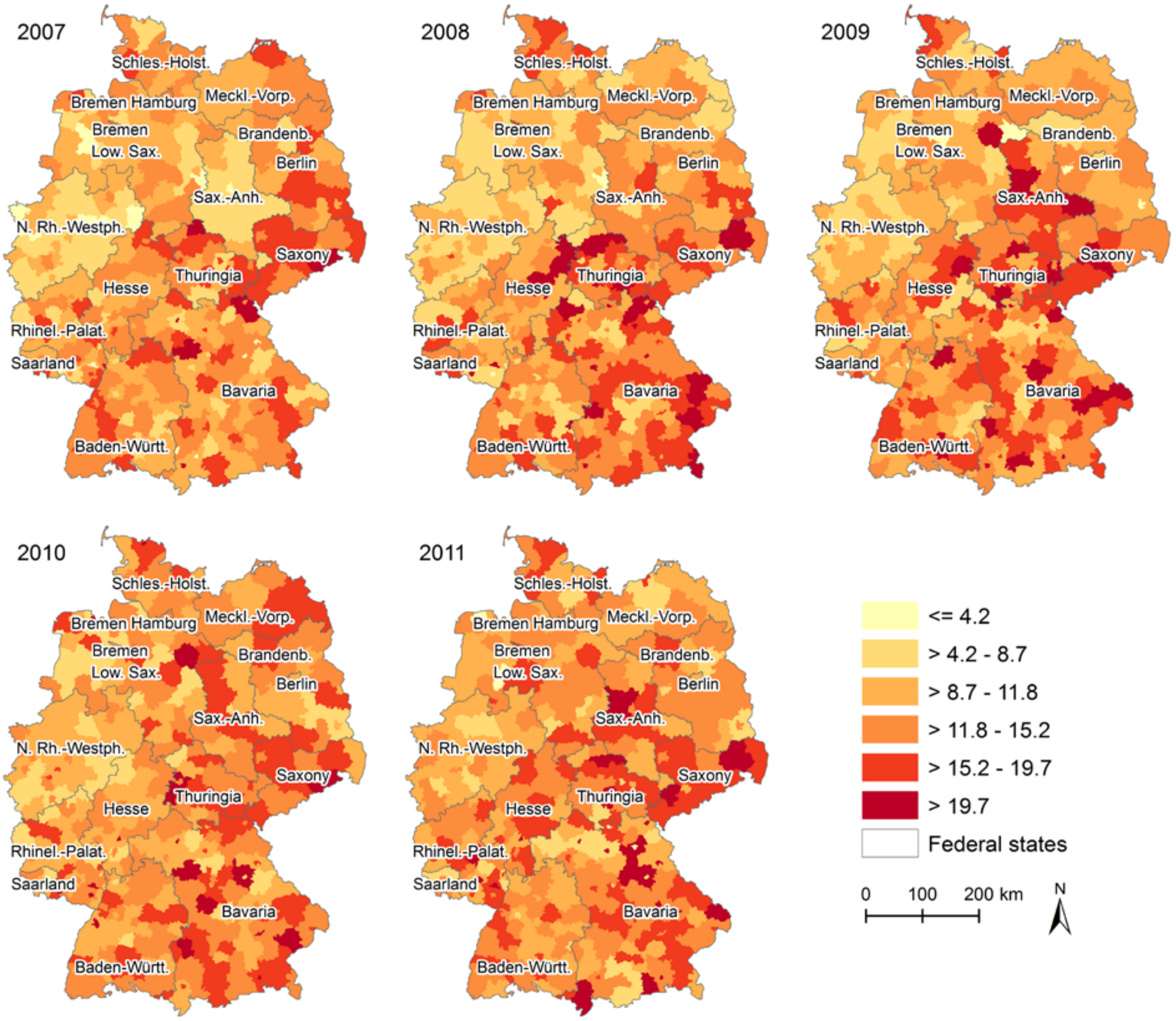

Supplementary Figure S2. Annual suicide rate per 100,000 persons at the district level (2007–11). Maps were created with ArcGIS 10.4.1 (www.esri.com).

Supplementary Table S3. Spearman correlations between the covariates.

2007	Income	Unemployment rate	Depression prevalence	General practitioners	Psychotherapists	Psychiatrists	Population density (log)
Income		-0.223	0.232	0.054	0.144	0.176	0.179
Unemployment rate	0.000		-0.260	-0.189	0.178	0.023	0.098
Depression prevalence	0.000	0.000		0.479	0.406	0.385	0.305
General practitioners	0.283	0.000	0.000		0.472	0.436	0.218
Psychotherapists	0.004	0.000	0.000	0.000		0.699	0.673
Psychiatrists	0.000	0.647	0.000	0.000	0.000		0.688
Population density (log)	0.000	0.050	0.000	0.000	0.000	0.000	
2008	Income	Unemployment rate	Depression prevalence	General practitioners	Psychotherapists	Psychiatrists	Population density (log)
Income		-0.216	0.221	0.049	0.125	0.164	0.170
Unemployment rate	0.000		-0.267	-0.188	0.186	0.045	0.125
Depression prevalence	0.000	0.000		0.479	0.406	0.385	0.306
General practitioners	0.327	0.000	0.000		0.472	0.436	0.219
Psychotherapists	0.012	0.000	0.000	0.000		0.699	0.673
Psychiatrists	0.001	0.372	0.000	0.000	0.000		0.689
Population density (log)	0.001	0.012	0.000	0.000	0.000	0.000	
2009	Income	Unemployment rate	Depression prevalence	General practitioners	Psychotherapists	Psychiatrists	Population density (log)
Income		-0.228	0.231	0.069	0.138	0.174	0.172
Unemployment rate	0.000		-0.244	-0.168	0.213	0.057	0.162
Depression prevalence	0.000	0.000		0.479	0.406	0.385	0.306
General practitioners	0.166	0.001	0.000		0.472	0.436	0.220
Psychotherapists	0.006	0.000	0.000	0.000		0.699	0.673
Psychiatrists	0.000	0.252	0.000	0.000	0.000		0.690
Population density (log)	0.001	0.001	0.000	0.000	0.000	0.000	
2010	Income	Unemployment rate	Depression prevalence	General practitioners	Psychotherapists	Psychiatrists	Population density (log)
Income		-0.177	0.235	0.069	0.140	0.180	0.177
Unemployment rate	0.000		-0.227	-0.181	0.233	0.084	0.212
Depression prevalence	0.000	0.000		0.479	0.406	0.385	0.307
General practitioners	0.167	0.000	0.000		0.472	0.436	0.221
Psychotherapists	0.005	0.000	0.000	0.000		0.699	0.673
Psychiatrists	0.000	0.093	0.000	0.000	0.000		0.691
Population density (log)	0.000	0.000	0.000	0.000	0.000	0.000	
2011	Income	Unemployment rate	Depression prevalence	General practitioners	Psychotherapists	Psychiatrists	Population density (log)
Income		-0.138	0.225	0.061	0.142	0.187	0.182
Unemployment rate	0.005		-0.225	-0.187	0.219	0.083	0.213
Depression prevalence	0.000	0.000		0.479	0.406	0.385	0.308
General practitioners	0.224	0.000	0.000		0.472	0.436	0.222
Psychotherapists	0.004	0.000	0.000	0.000		0.699	0.674
Psychiatrists	0.000	0.095	0.000	0.000	0.000		0.691
Population density (log)	0.000	0.000	0.000	0.000	0.000	0.000	

P-values are given in the lower diagonal

Supplementary Table S4. Univariate Moran’s *I* results.

Suicide rate	2007	2008	2009	2010	2011
Univariate Moran's *I*	0.222	0.273	0.231	0.137	0.077
P-value	1e-04	1e-04	1e-04	2e-04	0.009

Supplementary Table S5. Bivariate Moran’s *I* results.

Suicide rates (*t*, *t*+1)	2007-2008	2008-2009	2009-2010	2010-2011
Bivariate Moran's *I*	0.191	0.258	0.240	0.121
P-value	1e-04	1e-04	1e-04	1e-04

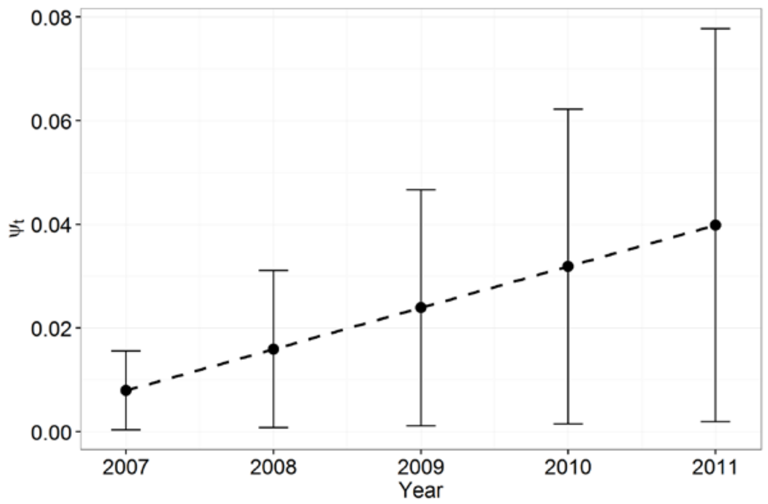

Supplementary Figure S6. Grand temporal trend with 95% credibility intervals.

References

1. Schrödle, B. & Held, L. Spatio-temporal disease mapping using INLA. *Environmetrics* **22,** 725–734 (2011).
2. Blangiardo, M., Cameletti, M., Baio, G. & Rue, H. Spatial and spatio-temporal models with R-INLA. *Spat. Spatiotemporal. Epidemiol.* **7,** 39–55 (2013).
3. Bernardinelli, L. *et al.* Bayesian analysis of space-time variation in disease risk. *Stat. Med.* **14,** 2433–2443 (1995).
4. Knorr-Held, L. Bayesian modelling of inseparable space-time variation in disease risk. *Stat. Med.* **19,** 2555–2567 (2000).
5. Ugarte, M. D., Goicoa, T., Ibanez, B. & Militino, A. F. Evaluating the performance of spatio-temporal Bayesian models in disease mapping. *Environmetrics* **20,** 647–665 (2009).
6. Kang, S. Y., McGree, J., Baade, P. & Mengersen, K. A case study for modelling cancer incidence using Bayesian spatio-temporal models. *Aust. N. Z. J. Stat.* **57,** 325–345 (2015).

7. Stack, S. Suicide: a 15-year review of the sociological literature part II: Modernization and social integration perspectives. *Suicide Life-Threatening Behav.* **30,** 163–176 (2000).
8. Congdon, P. Assessing the impact of socioeconomic variables on small area variations in suicide outcomes in England. *Int. J. Environ. Res. Public Health* **10,** 158–177 (2012).
9. Besag, J., York, J. & Mollié, A. Bayesian image restoration, with two applications in spatial statistics. *Ann. Inst. Stat. Math.* **43,** 1–20 (1991).
10. Lawson, A. B. *Bayesian disease mapping: hierarchical modeling in spatial epidemiology*. (CRC press, 2013).
11. Rue, H., Martino, S. & Chopin, N. Approximate bayesian inference for latent Gaussian models by using integrated nested Laplace approximations. *J. R. Stat. Soc. Ser. b* **71,** 319–392 (2009).
12. Sørbye, S. H. & Rue, H. Scaling intrinsic Gaussian Markov random field priors in spatial modelling. *Spat. Stat.* **8,** 39–51 (2014).
13. Spiegelhalter, D. J., Best, N. G., Carlin, B. P. & Linde, A. The deviance information criterion: 12 years on. *J. R. Stat. Soc. Ser. B (Statistical Methodol.* **76,** 485–493 (2014).
14. Pettit, L. I. The conditional predictive ordinate for the normal distribution. *J. R. Stat. Soc. Ser. B* 175–184 (1990).
